# On the Crystal Structural Control of Sputtered TiO_2_ Thin Films

**DOI:** 10.1186/s11671-016-1531-5

**Published:** 2016-07-07

**Authors:** Junjun Jia, Haruka Yamamoto, Toshihiro Okajima, Yuzo Shigesato

**Affiliations:** Graduate School of Science and Engineering, Aoyama Gakuin University, 5-10-1 Fuchinobe, Chuo Sagamihara, 252-5258 Japan; Kyushu Synchrotron Light Research Center, 8-7 Yayoigaoka, Tosu, Saga 841-0005 Japan

## Abstract

In this study, we focused on the origin on the selective deposition of rutile and anatase TiO_2_ thin films during the sputtering process. The observation on microstructural evolution of the TiO_2_ films by transmission electron microscopy revealed the coexistence of rutile and anatase TiO_2_ phases in the initial stage under the preferential growth conditions for the anatase TiO_2_; the observations further revealed that the anatase phase gradually dominated the crystal structure with increasing film thickness. These results suggest that the bombardment during the sputtering deposition did not obviously affect the TiO_2_ crystal structure, and this was also confirmed by off-axis magnetron sputtering experiments. We also investigated the mechanism of the effect of Sn impurity doping on the crystal structure using first-principles calculations. It is found that the formation energy of Sn-doped rutile TiO_2_ is lower than that of Sn-doped anatase TiO_2_; this suggests that the Sn-doped TiO_2_ favours the rutile phase. These results offer a guideline for the utilization of selective deposition of rutile and anatase TiO_2_ thin films in various industrial applications.

## Background

Rutile and anatase TiO_2_ films are widely used in various industrial applications [1–3]. For example, rutile TiO_2_ films are used as an optical coating material because of their high refractive index whereas anatase TiO_2_ films are utilised as photocatalysts or transparent electrodes [3]. Rutile TiO_2_ is the most common phase in nature, and anatase TiO_2_ transforms to rutile at temperatures above 400–600 °C [4].

Conventional wet processes such as the sol–gel method can be used to produce pure-phase TiO_2_ films; however, fabricating dense TiO_2_ films is difficult by this method [5]. Sputtering deposition can be used to produce uniform TiO_2_ thin films with a large area, high packing density and strong adhesion [1, 6]. However, TiO_2_ films deposited by magnetron sputtering are often a mixture of anatase and rutile phases. As a practical measure, controlling the phase content of TiO_2_ films is necessary for films used in precise optical applications. Therefore, the deposition of pure-phase TiO_2_by magnetron sputtering has attracted much attention. Currently, two approaches can be used to fabricate pure-phase sputtered TiO_2_ films. One is to control the sputtering conditions such as the total gas pressure, substrate temperature and type of sputtering gas to selectively fabricate uniform coatings of rutile or anatase TiO_2_ films [1]; the other is to use impurity doping to induce a phase transformation between the anatase and rutile phases [1, 7–14].

In the sputtering process, rutile and anatase TiO_2_ films are easily fabricated under low and high total gas pressure, respectively [1, 15, 16]. A general explanation for this observation is that bombardment by high-energy particles such as negative oxygen ions can lead to a dense rutile TiO_2_ phase [1]. However, the bombardment effects have not been confirmed experimentally, and the mechanism by which the rutile or anatase TiO_2_ phase grows during the sputtering process remains unknown. In the case of impurity doping, elements such as Mn [7], Fe [7], Cu [7], Ag [8], Ni [9] and Co [9] have been reported to enhance the phase transition from the anatase to the rutile phase, whereas other elements such as W [7], V [10], Si [11], Nb [12], Ta [12] and Cr [13] have been reported to suppress the anatase-to-rutile phase transition. On the basis of first-principles calculations, the room-temperature phase conversion of anatase to rutile TiO_2_ using Co or Ni doping is attributed to the increased interaction between Co and Ni atoms, which results in the formation of a linear chain in the rutile phase [14]. In a previous study, we demonstrated that Sn doping can induce the anatase-to-rutile transformation in a sputtered TiO_2_ film [1]. However, the related mechanism for the transformation induced by Sn doping has not yet been elucidated.

In this study, we first use transmission electron microscopy (TEM) to observe the microstructural evolution of TiO_2_ films during sputtering. Second, we investigate the bombardment effects of high-energy particles on the crystal structure of TiO_2_ films using the off-axis sputtering method and discuss the effect of sputtered Ti particles on the crystal structure. Finally, we reveal the origin of the Sn-doping-induced anatase-to-rutile phase transformation on the basis of first-principles calculations.

## Methods

### Experimental Details

To investigate the microstructural evolution of the TiO_2_ films, TiO_2_ films with thicknesses of 50, 100, 200 and 500 nm were deposited by rf magnetron sputtering using a 3-in. diameter Ti metal target (99.99 %, Furu-uchi Kagaku), where the sputtering gas was Ar and the total gas pressure was set to 3.0 Pa. Moreover, we changed the sputtering gas from Ar to Kr or Ne to study the effect of sputtered Ti particles on the crystal structure at different total gas pressures (0.5, 1.0, 2.0 and 3.0 Pa). The oxygen flow ratio (O_2_/(Ar + O_2_)) was maintained at 60 %. The distance between the target and the substrate was 55 mm.

**Fig. 1:**
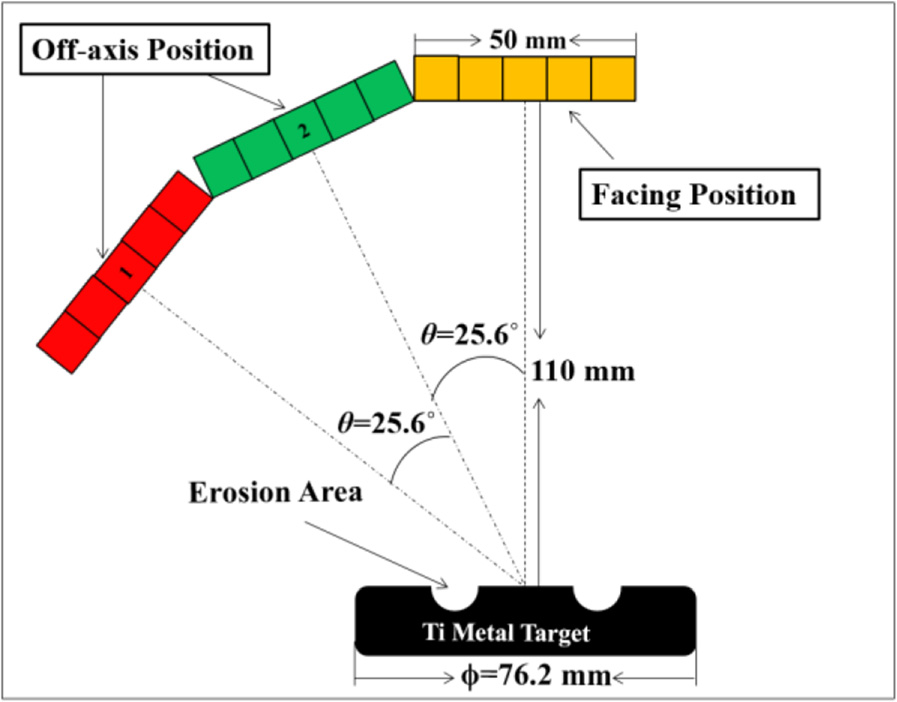
Schematic of off-axis magnetron sputtering

To investigate the bombardment effects on the crystal structure, we deposited TiO_2_ films onto unheated quartz substrates by off-axis dc magnetron sputtering. The configuration of the off-axis sputtering system is shown in Fig. 1 [17]. The distance between the target and the substrate was 110 nm. The sputtering power was maintained at 200 W, and pure Ar gas was used as the sputtering gas. The total gas pressure was set to 0.3 Pa. The O_2_ flow ratios (O_2_/(Ar + O_2_)) were set to 0, 20, 40 and 60 %.

The film thickness was measured using a surface profiler (Dektak^3^, Sloan Tech). X-ray diffraction (XRD, XRD-6000, Shimadzu) analysis was performed using Cu K_α1_ radiation generated at 40 kV and 20 mA. Microstructural studies were performed using TEM (JEM-4010, JEOL).

Sn K-edge X-ray absorption fine structure (XAFS) spectra of the Sn-doped TiO_2_ thin films was measured at beamline BL07 of the SAGA Light Source [18] using convergent electron yield (CEY) mode. We also collected the spectra of 0.05-mm-thick Sn foil and SnO and SnO_2_ powders diluted with high-purity hexagonal BN powder as reference samples using transmission mode. All the measurements were conducted in air at room temperature.

### Theoretical calculations

In our previous study, we used X-ray absorption near edge structure (XANES) spectra to demonstrate that Sn doping induces the anatase-to-rutile phase transformation in sputtered TiO_2_ films. In the present study, we used first-principles calculations to investigate the geometrical structure of Sn doping and the transformation mechanism from anatase to rutile.

All the first-principles calculations were based on a plane-wave pseudopotential method using the CASTEP code [19, 20]. Vanderbilt ultrasoft pseudopotentials were employed, and the generalised gradient approximation (GGA-PBE) [21] was used as an exchange-correlation functional. After careful convergence tests with respect to the number of k-points and the plane-wave cutoff, a Monkhorst–Pack k-point grid with a special resolution of 0.5 nm^−1^ and a plane-wave cutoff energy of 380 eV was used for all calculations.

**Fig. 2:**
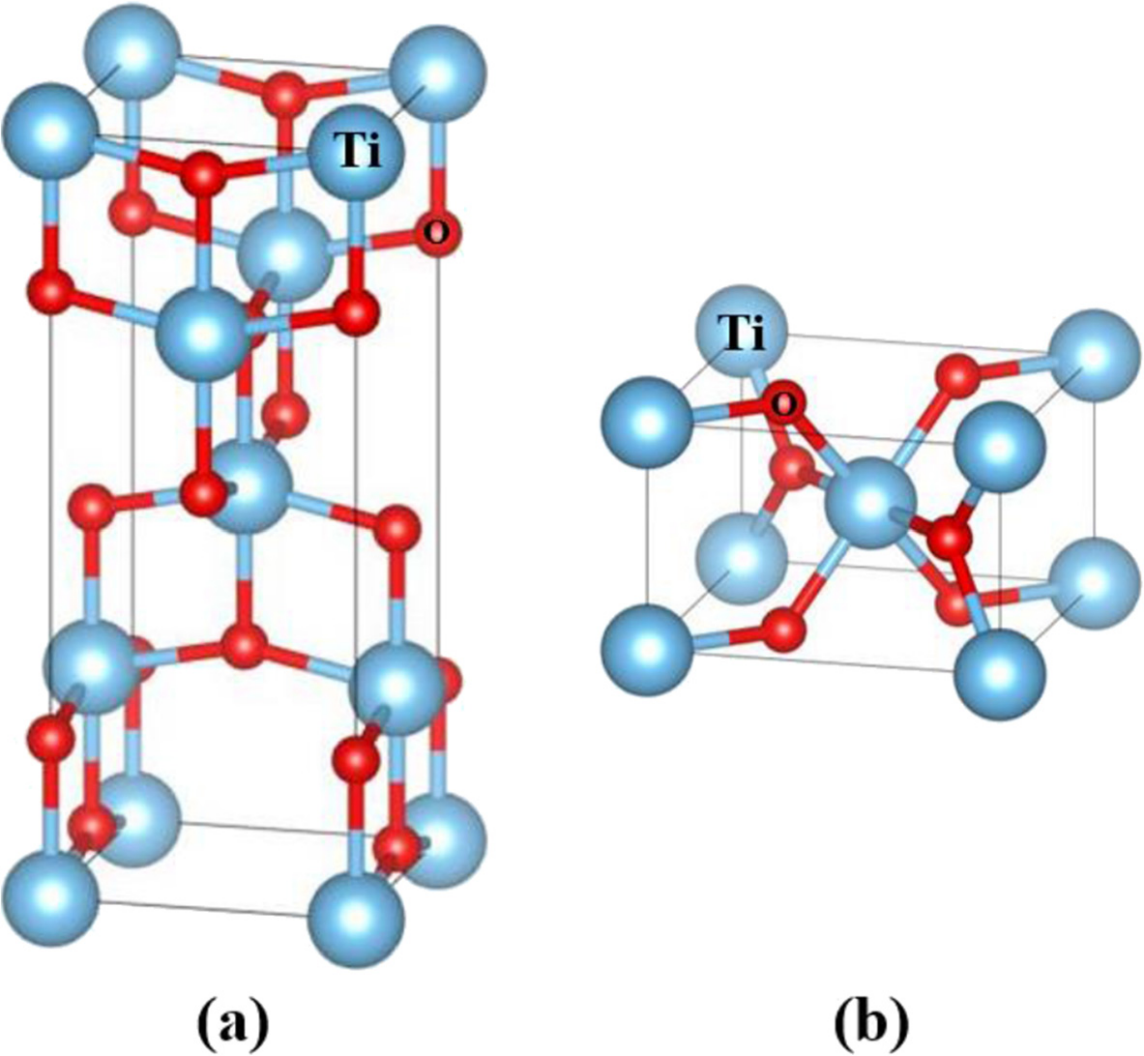
Unit cells of anatase TiO_2_ (**a**) and rutile TiO_2_ (**b**). *Large light-blue* and *small red spheres* are Ti^4+^ and O^2−^ ions, respectively

**Fig. 3:**
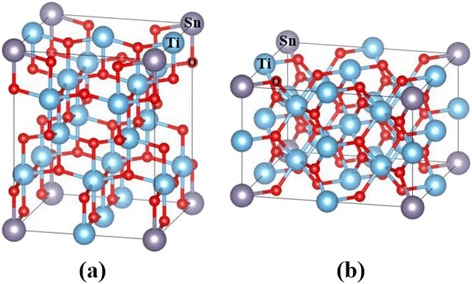
Sn-doped 2 × 2 × 1 supercell of anatase TiO_2_ (**a**) and 2 × 2 × 2 supercell of anatase TiO_2_ (**b**). *Large purple circles* are Sn^4+^ ions

Figure 2 shows the geometrical structures of the unit cells of anatase and rutile TiO_2_. The experimentally reported tetragonal (*I*4_1_/*amd*) [22] and tetragonal (*P*4_2_/*mnm*) [23] structures for anatase and rutile TiO_2_ were adopted as initial structures, respectively. The unit cells contain four Ti atoms and eight O atoms for anatase TiO_2_ and two Ti atoms and four O atoms for rutile TiO_2_, respectively. In the calculations, a supercell consisting of four unit cells in 2 × 2 × 1 configuration for anatase TiO_2_ and eight unit cells in 2 × 2 × 2 configuration for rutile TiO_2_ of the optimised unit cell (48 atoms) was employed, as shown in Fig. 3. On the basis of our previous results, that is, Sn^4+^ ions doped into TiO_2_ and substituted into Ti^4+^ sites, one Ti atom in the supercells was substituted by a Sn atom. The atomic concentration of the Sn atom was 6.25 %. In all of the calculations, the internal atomic positions of the atoms in the cells were allowed to relax with a fixed size of the supercell, i.e. the shape of the supercell was fixed at a theoretically optimised shape for pure anatase and rutile TiO_2_ under the assumption of conditions at the dilute limit.

## Results and Discussion

### TEM Observation of Thin-Film Growth

**Fig. 4:**
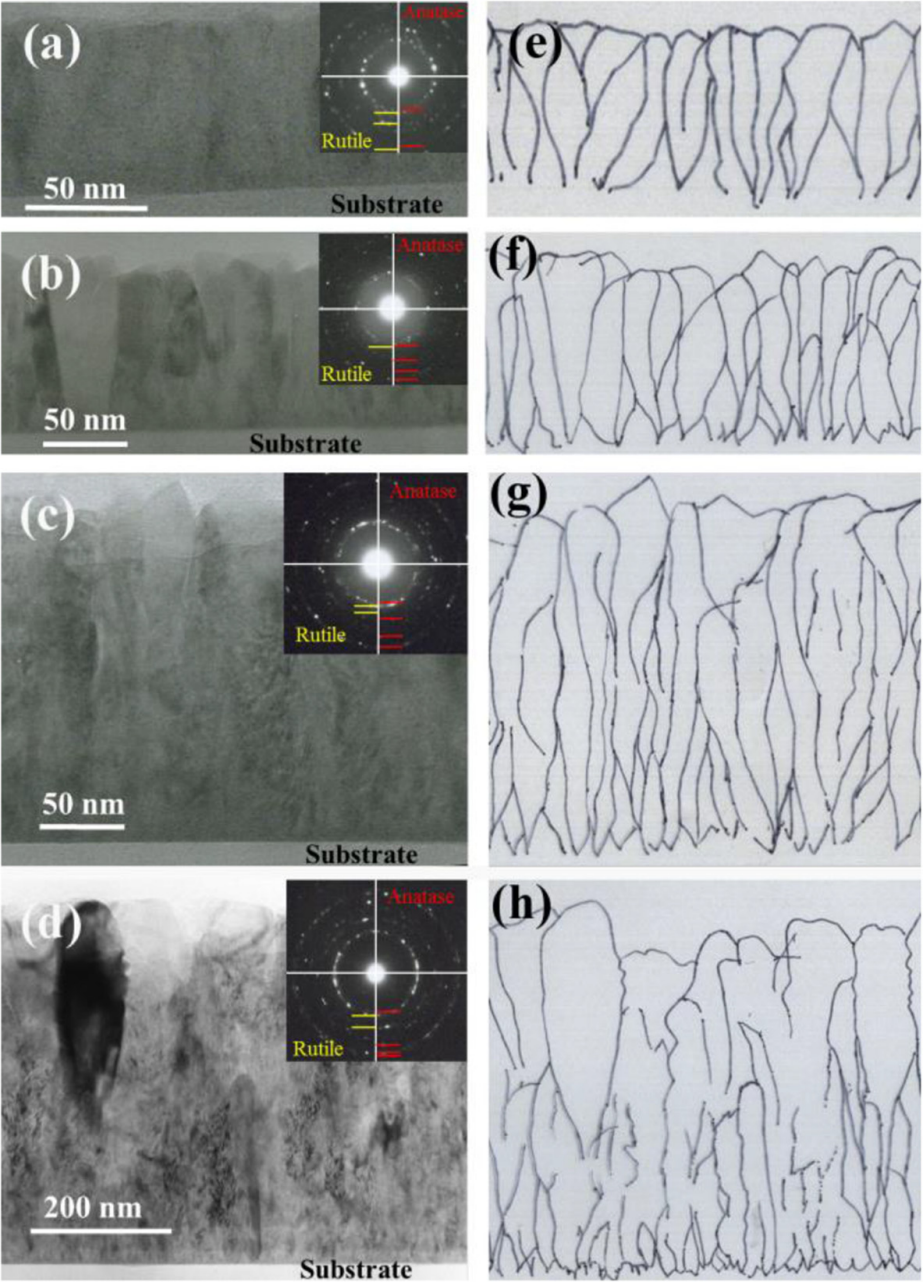
Cross-sectional bright-field TEM images of TiO_2_ films with thicknesses of 50 nm (**a**), 100 nm (**b**), 200 nm (**c**) and 500 nm (**d**). **e**–**h** The corresponding sketch images of the cross-sectional microstructures. The *insets* are the selected-area electron diffraction patterns

TEM images of TiO_2_ films with thicknesses of 50, 100, 200 and 500 nm, which were deposited at a total gas pressure of 3.0 Pa in the facing positions, are presented in Fig. 4. Figure 4a–d shows cross-sectional bright-field TEM images of TiO_2_ films with thicknesses of 50 nm (a), 100 nm (b), 200 nm (c), and 500 nm (d). Figure 4e–h shows the corresponding sketch images of their cross-sectional microstructures. The cross-sectional TEM images clearly show the columnar polycrystalline structure of typical films deposited by sputtering. The sketch images demonstrate that many small crystallites were formed on the substrate in the initial growth stage and that these crystallites gradually decreased in number and increased in grain size with increasing film thickness; we attributed this behaviour to the growth competition among the crystallites [24].

**Fig. 5:**
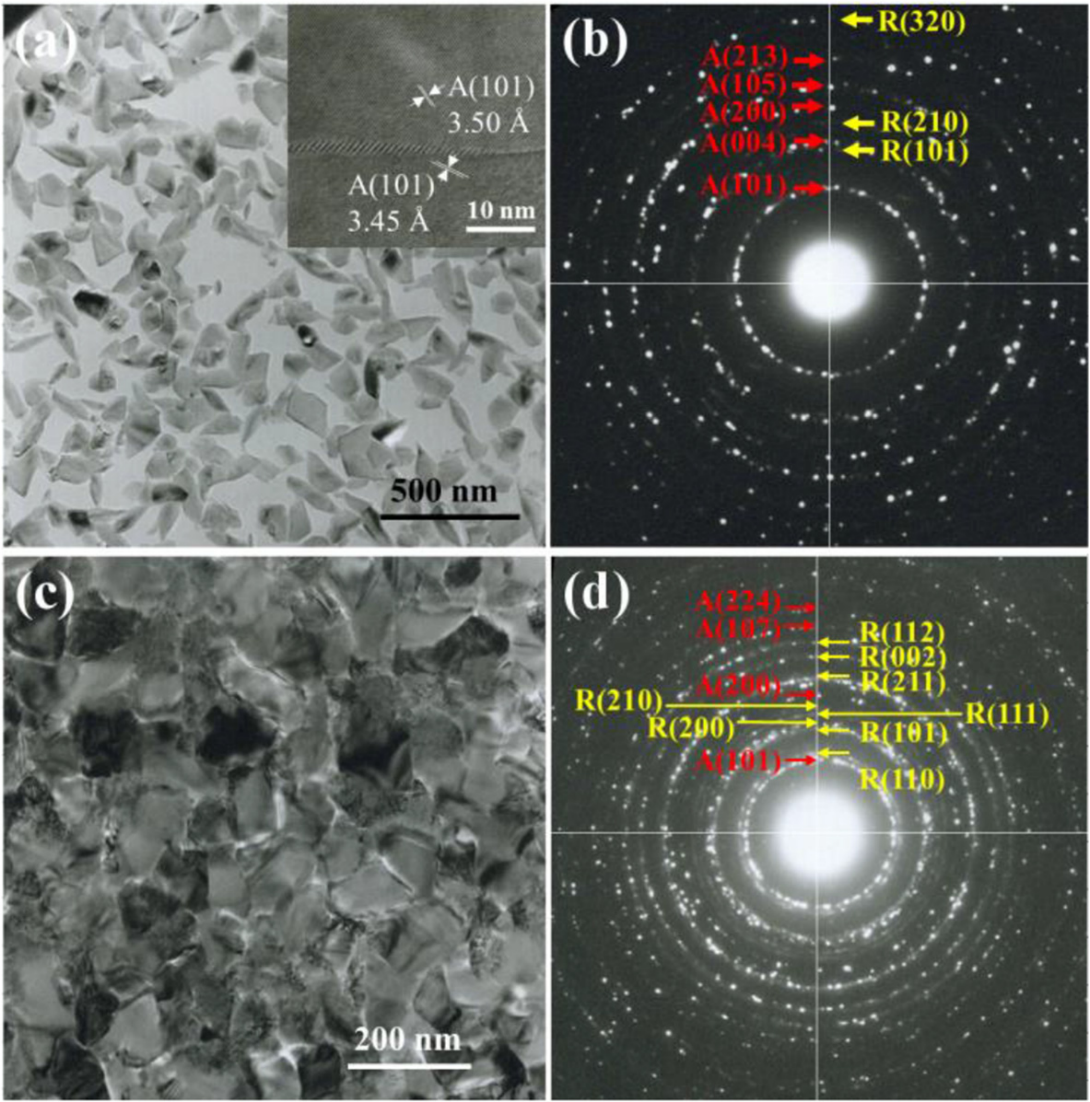
**a**, **b** Plane-view TEM images and electron diffraction pattern, respectively, at the surface region of TiO_2_ films with a thickness of 500 nm; **c**, **d** Plane-view TEM images and electron diffraction pattern near the substrate of a TiO_2_ film with a thickness of 500 nm, respectively

The Debye–Scherrer rings in the electron diffraction patterns (inset in Fig. 4) suggest the coexistence of rutile and anatase phases in TiO_2_ films. To investigate the distribution of anatase and rutile phases along the thickness direction, we conducted plane-view TEM analyses of the TiO_2_ film with the thickness of 500 nm at both the surface region and the bottom region (near the substrate). Figure 5a, b shows the plane-view images and electron diffraction patterns, respectively, of the TiO_2_ film at the surface region, whereas Fig. 5c, d shows images in the bottom region (near the substrate). The diffraction patterns indicate that the anatase and rutile phases coexist in the bottom region, whereas the anatase phase dominates the crystal structure in the surface region.

On the basis of the aforementioned experimental observations, we concluded that the anatase and rutile phases coexist during the initial growth stage and that the anatase phase gradually dominates the crystal structure to form large crystallites with increasing film thickness. These films were deposited at a total gas pressure of 3.0 Pa, which is the preferential growth condition for anatase TiO_2_ films in the sputtering process, as shown in Fig. 7. In our previous report, we attributed the formation of anatase TiO_2_ films at a total gas pressure of 3.0 Pa to the suppressed bombardment effect from the high-energy particles due to gas scattering. However, such an explanation appears to be inconsistent with the present TEM observations that the rutile phase is still observed during the initial growth stage even under the anatase-preferential growth conditions because, in principle, the bombardment from the high-energy particles should homogeneously affect the film in the direction of the film thickness. Thus, the bombardment effect may not be a main factor for the formation of rutile phase during the sputtering process (we subsequently confirmed the weak influence of the bombardment effect by off-axis sputtering, as discussed in the following section). Such a growth behaviour can likely be attributed to the strong thermodynamic driving force towards the *I*4_1_/*amd* phase, which results in smaller critical radii of the crystal nuclei and accelerates nucleation over the growth process [12].

### Bombardment Effect

Because the rutile phase is more dense (4.250 g · cm^−3^) than the anatase phase (3.894 g · cm^−3^), the rutile phase is considered to be stable at both high temperatures and high pressures [4, 25]. The bombardment effect of high-energy particles during the sputtering process has been reported to lead to a dense film [26]. Thus, the bombardment effect is considered a possible reason for the production of rutile phase. In this study, off-axis sputtering was used to confirm this possible mechanism. In general, negative oxygen ions are considered to be the origin of the bombardment effect because of their high energy (approximately several hundred electron volts) [6]. In the sputtering process, negative oxygen ions are accelerated by the cathode sheath and move towards the substrate in a straight path; thus, the bombardment effect from the high-energy particles should be observed in the facing position, as shown in Fig. 1. Consequently, more rutile phase is expected to be produced in the facing position.

**Fig. 6:**
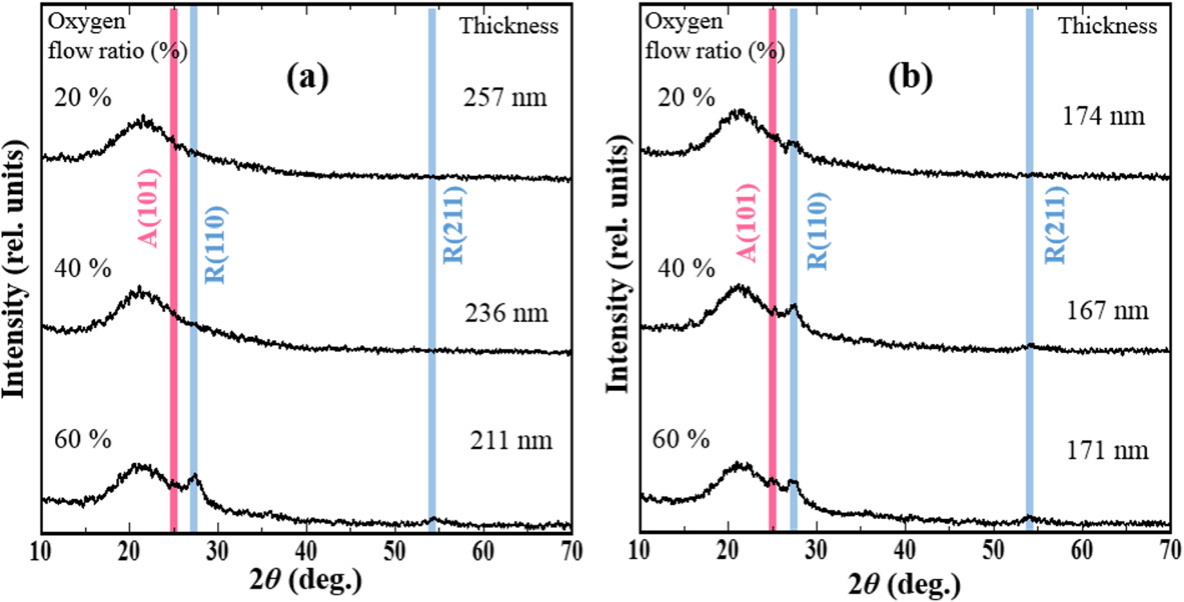
XRD patterns of TiO_2_ films deposited at the off-axis position 1 (**a**) and facing positions (**b**) under various oxygen flow ratios. The *pink* and *blue lines* represent the peaks from anatase and rutile TiO_2_, respectively

Figure 6 shows the XRD spectra of TiO_2_ films prepared under 0.3 Pa using off-axis dc magnetron sputtering. The peaks at 2*θ* ≈ 25.28° are attributed to the (101) plane of the anatase phase, and the peaks at 2*θ* ≈ 27.45° are attributed to the (110) plane of the rutile phase. The anatase phase was observed in the facing position, and the rutile phase was observed in the off-axis positions. These results imply that the bombardment did not obviously affect the crystal structure of the sputtered TiO_2_ films.

Figure 6 also shows the XRD patterns of different deposition locations under various oxygen flow ratios. The XRD patterns suggest that the intensity of rutile TiO_2_ peaks in the facing position increases with increasing oxygen flow ratio. Raman measurements show that the proportion of rutile TiO_2_ phase increases with increasing oxygen flow ratio for TiO_2_ films deposited in the facing position and off-axis positions (data not shown). Another interesting phenomenon is that the deposition rate depends on both the oxygen flow ratio and the deposition locations, as reported in Table 1; this effect is attributable to a certain number and energy angular distribution of the sputtered particles from the target [27]. When the oxygen flow ratio is 0 %, the deposition rate in the off-axis position is obviously faster than that in the facing position. With increasing oxygen flow ratio, the difference in deposition rate between the off-axis position and the facing position becomes unobvious. This result implies that the TiO_2_ film deposited in the off-axis position is in a relatively reduced state compared to those deposited in the facing position.

**Table 1:** Deposition rate (nm · min^−1^) of TiO_2_ films at different oxygen flow ratios

O_2_ flow ratio	0 %	20 %	40 %	60 %
Off-axis position 1	17.5	1.62	0.98	1.03
Off-axis position 2	15.4	1.20	0.78	0.92
Facing position	11.9	1.09	0.70	0.83

### Energy of Sputtered Ti particles

**Fig. 7:**
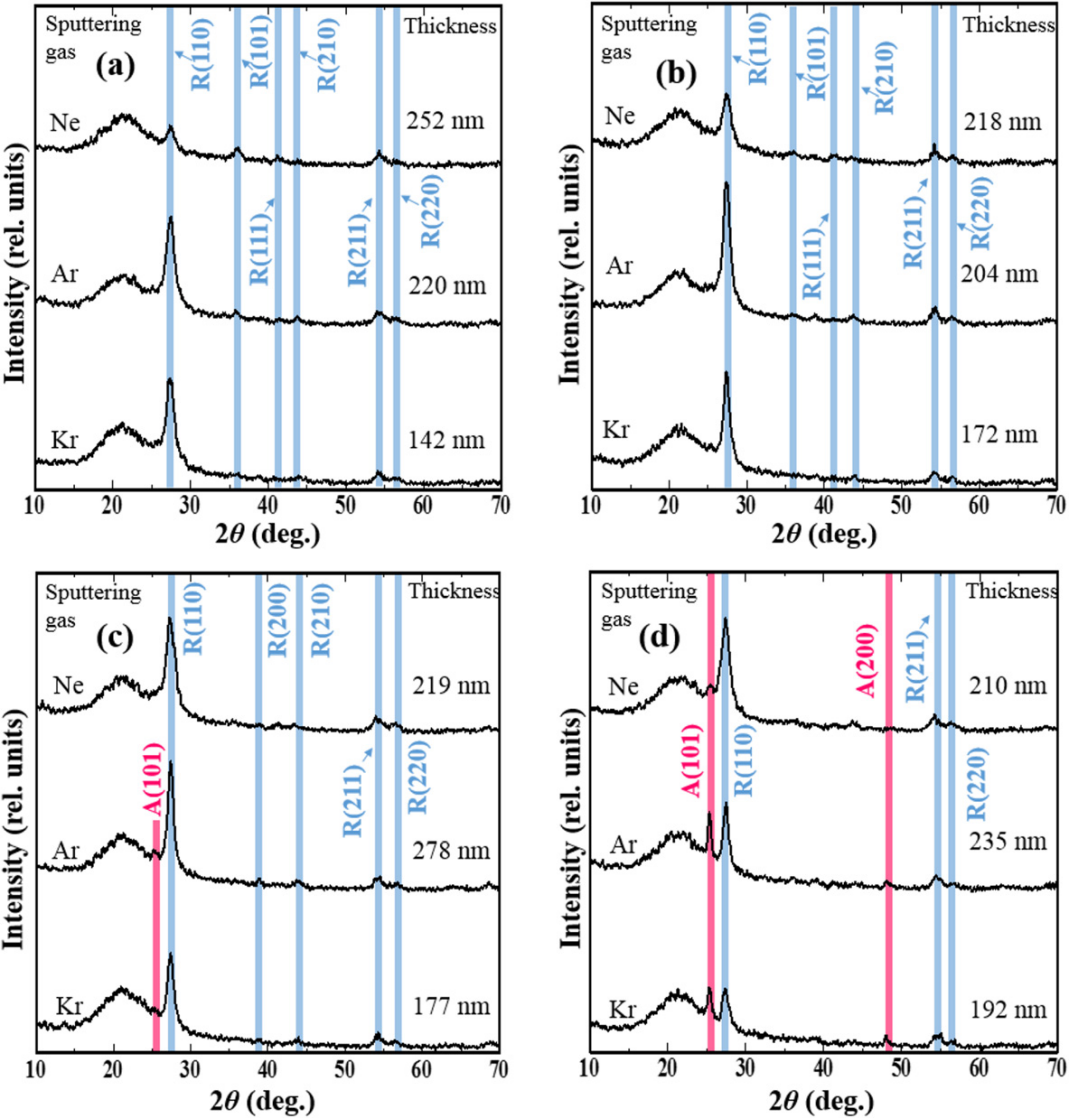
XRD patterns of TiO_2_ films deposited under different sputtering gases and different total gas pressures: 0.3 Pa (**a**), 1.0 Pa (**b**), 2.0 Pa (**c**) and 3.0 Pa (**d**). The *pink* and *blue lines* represent the peaks from anatase and rutile TiO_2_, respectively. The oxygen flow ratio was set to 60 %

The kinetic energy of sputtered Ti particles also affects the crystal structure of TiO_2_ films [1]. In this study, we changed the sputtering gases (Ar, Kr and Ne) to vary the kinetic energy of sputtered Ti particles. Figure 7a–d shows the XRD patterns of TiO_2_ films deposited under different sputtering gases and total gas pressures. The rutile TiO_2_ phase was observed at all investigated total gas pressures. When the total gas pressure was greater than 2.0 Pa, the diffraction peak from the rutile phase gradually decreased in intensity in the XRD patterns of films deposited under all sputtering gases, and anatase phase was observed in the patterns of films deposited under Ar and Kr sputtering gases. When the total pressure was increased to 3.0 Pa, anatase TiO_2_ was observed in the case of Ne sputtering gas.

**Fig. 8:**
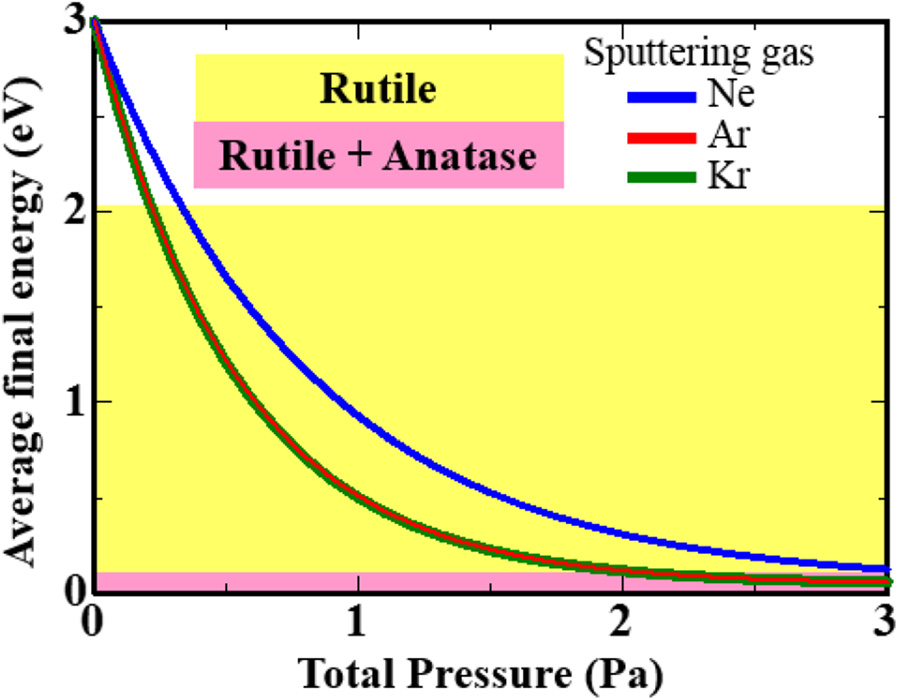
Final kinetic energy of sputtered Ti particles reaching the growing film surface, as estimated by the equation from Meyer et al. The *yellow region* shows the pure rutile phase, whereas the *pink region* represents the coexistence of anatase and rutile phases

To summarise these experimental results, we estimated the energy distribution of sputtered particles using the equation of Meyer et al. [28]. A change in the sputtering gas or the total gas pressure led to a change of the kinetic energy of the sputtered Ti particles because of gas scattering resulting from collisions with gas molecules. The energy of sputtered Ti reaching the growing film surface was estimated using an equation from Meyer et al.:

1 $$ {E}_{\mathrm{F}}=\left({E}_0-{k}_{\mathrm{B}}{T}_{\mathrm{G}}\right) \exp \left\{Nln\left(\frac{E_1}{E_2}\right)\right\}+{k}_B{T}_G $$

where *E*_0_ and *E*_F_ are the initial and final energies of Ti, respectively, *k*_B_ is the Boltzmann constant, *T*_G_ is the temperature of the sputtering gas, *N* is the collision number, and *E*_1_/*E*_2_ is the ratio of energy before and after a collision. Here *N* and *E*_1_/*E*_2_ are given by

2 $$ N=\frac{d{p}_{\mathrm{tot}}\sigma }{k_B{T}_G} $$

and

3 $$ {E}_1/{E}_2=1-\frac{2y}{{\left(1+y\right)}^2} $$

where *d* is the distance travelled, *σ* is the collision cross-section (assuming hard core interactions), and *γ* is the atomic mass ratio of collision particles. Figure 8 shows the calculated final kinetic energies for the sputtered Ti particles reaching the substrate as a function of the total gas pressure under different sputtering gases. The *E*_0_ of the sputtered Ti was assumed to be 3 eV [15]. When the kinetic energy of sputtered Ti particles was between 0.1 and 0.2 eV, the anatase phase was observed. From the viewpoint of thermal equilibrium, the average energy of 0.1 eV is related to a temperature of 500 °C, which is similar to the transition temperature (600 °C) from anatase to rutile in bulk TiO_2_ [4]. These results imply that the final kinetic energy of sputtered Ti particles may be important for the selective deposition of rutile or anatase phase in the sputtering process.

### Mechanism of Sn-Doping-Induced Transformation from Anatase to Rutile

In addition to control of the sputtering conditions, impurity doping is an effective method of controlling the crystal structure of the sputtered TiO_2_ films. Li et al. attributed the room-temperature phase conversion of anatase to rutile TiO_2_ using Co or Ni to increased interaction between Co and Ni atoms forming a linear chain in the rutile phase [14]. In our previous reports, we observed that impurity Sn doping also induced the phase transformation from the anatase to the rutile phase in sputtered TiO_2_ films [1]. The XANES spectra suggest that Sn^4+^ ions are doped into Ti sites in TiO_2_ films. However, the geometrical doping structure of the Sn dopant and its effect on the growth of the crystal structure remain unknown.

**Fig. 9:**
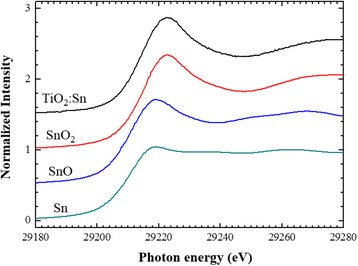
Observed Sn K-edge XANES spectrum of a Sn-doped TiO_2_ film with a Sn content of 37.6 at.%; the spectra of reference samples (Sn, SnO, SnO_2_) are included for comparison

**Fig. 10:**
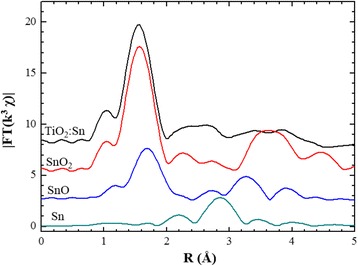
Magnitude of the Fourier-transformed EXAFS (k^3^χ) spectra of a Sn-doped TiO_2_ film with a Sn content of 37.6 at.% and the reference samples. The FTs were processed with a k-range of 2.5–10 Å^ − 1 ^for Sn K-edge EXAFS spectra

Figure 9 shows the XANES spectrum of Sn K-edge for a Sn-doped TiO_2_ thin film with a Sn content of 37.6 at.%; the spectra of reference samples are also shown. An intense peak is observed at approximately 29,220 eV in all of the spectra. The peak positions are monotonically shifted towards higher energy as a function of the Sn valence. This result shows that the Sn in the TiO_2_ film exists as tetravalent Sn^4+^ ions. The magnitude of the Fourier-transformed EXAFS from the Sn K-edge EXAFS spectra of the Sn-doped TiO_2_ thin film with a Sn content of 37.6 at.% and the reference samples are shown in Fig. 10 as a function of the phase-uncorrected interatomic distance *R*. SnO_2_ and SnO have tetragonal (*P*4_2_/*mnm*) [23] and tetragonal (*P*4/*nmm*) [29] structures, respectively. The structure of SnO_2_ is the same as that of rutile TiO_2_. An intense peak is observed at approximately 1.6 Å in the spectra of both Sn-doped TiO_2_ and SnO_2_, at approximately 1.7 Å in the spectrum of SnO and at approximately 2.8 Å in the spectrum of Sn foil. The intense peak observed in the SnO_2_ and SnO spectra corresponds to the atomic distance between a Sn atom and its first-nearest-neighbour O atoms. By contrast, the intense peak observed in the spectrum of Sn foil corresponds to the distance of between a Sn atom and its first-nearest-neighbour Sn atoms. The peak position and spectral features of the Sn-doped TiO_2_ thin film are similar to those of rutile-structured SnO_2_, indicating that the local structures of Sn atoms in TiO_2_ are similar to those of SnO_2_. Table 2 shows the results of fitting analysis of Sn-doped TiO_2_ and SnO_2_ using the Artemis software package, which was used to analyse the EXAFS data [30]. In the fitting, the coordination numbers of the first-nearest-neighbour oxygen atoms were fixed at 6. The interatomic distance between Sn and O was determined to be 2.04 Å, which approaches the value in rutile SnO_2_ [31]. These results also indicate that a Sn atom with 4+ valence was substituted into a Ti site in the rutile TiO_2_.

**Table 2:** First-shell EXAFS fitting results for the Sn–O direct bond in a Sn-doped TiO_2_ film and in a SnO_2_ reference sample. In these fittings, the coordination number was fixed at 6. Here, *r* is the phase-corrected atomic distance and *σ* ^2^ is the Debye–Waller disorder factor

Sample	*r* (Å)	*σ* ^2^ (Å^2^)	*R* factor
Sn-doped TiO_2_	2.04 (3)	0.002 (4)	0.00343
SnO_2_	2.05 (4)	0.002 (3)	0.00354

To demonstrate the relative stability of the models, we compared the formation energy of Sn-doped TiO_2_ for both anatase and rutile TiO_2_ by first-principles calculations. The formation energies, *E*^F^, were obtained from the following equation:

4 $$ {E}^F={E}_t\left[T{i}_{15}{\mathrm{SnO}}_{32}\right]-{E}_t\left[{\mathrm{Ti}}_{16}{\mathrm{O}}_{32}\right]+{E}_t\left[{\mathrm{Ti}\mathrm{O}}_2\right]-{E}_t\left[{\mathrm{SnO}}_2\right] $$

where *E*_t_ is the total energy of the unit cell of TiO_2_ and SnO_2_ and the super supercell with/without the dopant. The total energies of TiO_2_ (*I*2_1_/*amd*) [22] and SnO_2_ (*P*4_2_/*mnm*) [23] were obtained after their crystal structures were optimised by the same computational methods previously described. The obtained formation energies are 0.66 and 0.25 eV for anatase and rutile TiO_2_, respectively. The formation energy for rutile TiO_2_ is lower than that for anatase TiO_2_ by 0.41 eV. The rutile TiO_2_ is more suitable for the Sn-doped TiO_2_ system. That is, Sn-doped TiO_2_ favours the rutile phase. This conclusion is consistent with that from our previous experimental report.

## Conclusions

In this study, TiO_2_ films were prepared on unheated glass substrates using dc off-axis and rf magnetron sputtering methods, and the mechanism of the selective deposition of rutile and anatase TiO_2_ films during the sputtering process was investigated. TEM observations of the microstructural evolution of the TiO_2_ films showed the coexistence of rutile and anatase TiO_2_ phases in the initial stage, even in under anatase-preferential growth conditions; the anatase phase gradually dominated the crystal structural with increasing film thickness. These results suggest that the bombardment had no obvious effect on the TiO_2_ crystal structure during the sputtering process, which was also confirmed by off-axis magnetron sputtering experiments. Moreover, we studied the relationship between the kinetic energy of sputtered Ti particles and the crystal structure of TiO_2_ films and observed that the anatase TiO_2_ thin film was easily formed when the kinetic energy of sputtered Ti particles was less than 0.1 eV.

The mechanism of the effect of Sn impurity doping on the crystal structure was investigated by first-principles calculations. We observed that the formation of Sn-doped rutile TiO_2_ was lower than that of Sn-doped anatase TiO_2_, suggesting that Sn-doped TiO_2_ favours the rutile phase. These results offer a guideline for the selective deposition of rutile and anatase TiO_2_ thin films for industrial applications.
